# Bone Regeneration Capacity of Newly Developed Uncalcined/Unsintered Hydroxyapatite and Poly-l-lactide-co-glycolide Sheet in Maxillofacial Surgery: An In Vivo Study

**DOI:** 10.3390/nano11010022

**Published:** 2020-12-24

**Authors:** Huy Xuan Ngo, Quang Ngoc Dong, Yunpeng Bai, Jingjing Sha, Shinji Ishizuka, Tatsuo Okui, Shintaro Sukegawa, Takahiro Kanno

**Affiliations:** 1 Department of Oral and Maxillofacial Surgery, Shimane University Faculty of Medicine, Izumo, Shimane 693-8501, Japan; ngoxuanhuy158@gmail.com (H.X.N.); dongngocquang1987@gmail.com (Q.N.D.); xyywq@126.com (Y.B.); jsswjbnjw@gmail.com (J.S.); ishizuka@med.shimane-u.ac.jp (S.I.); tokui@med.shimane-u.ac.jp (T.O.); 2 Division of Oral and Maxillofacial Surgery, Kagawa Prefectural Central Hospital, Takamatsu, Kagawa 760-8557, Japan; s-sukegawa@chp-kagawa.jp

**Keywords:** bone regeneration, leptin receptor, osteocalcin, osteoconductivity, poly-l-lactide-co-glycolide, poly-l-lactic acid, Runx2, uncalcined/unsintered hydroxyapatite

## Abstract

Uncalcined/unsintered hydroxyapatite and poly-l-lactide-co-glycolide (u-HA/PLLA/PGA) is a new bioresorbable nanomaterial with superior characteristics compared with current bioresorbable materials, including appropriate mechanical properties, outstanding bioactive/osteoconductive features, and remarkably shorter resorption time. Nevertheless, the bone regeneration characteristics of this nanomaterial have not been evaluated in maxillofacial reconstructive surgery. In this study, we used a rat mandible model to assess the bone regeneration ability of u-HA/PLLA/PGA material, compared with uncalcined/unsintered hydroxyapatite and poly-l-lactide acid (u-HA/PLLA) material, which has demonstrated excellent bone regenerative ability. A 4-mm-diameter defect was created at the mandibular angle area in 28 Sprague Dawley male rats. The rats were divided into three groups: u-HA/PLLA/PGA (u-HA/PLLA/PGA graft + defect), u-HA/PLLA (u-HA/PLLA graft + defect), and sham control (defect alone). At 1, 3, 8, and 16 weeks after surgeries, the rats were sacrificed and assessed by micro-computed tomography, histological analysis with hematoxylin and eosin staining, and immunohistochemical analyses. The results confirmed that the accelerated bone bioactive/regenerative osteoconduction of u-HA/PLLA/PGA was comparable with that of u-HA/PLLA in the rat mandible model. Furthermore, this new regenerative nanomaterial was able to more rapidly induce bone formation in the early stage and had great potential for further clinical applications in maxillofacial reconstructive surgery.

## 1. Introduction

In the late 20th century, bioresorbable polymers used to make bone fixation devices began to receive considerable attention in material research. Compared with their titanium counterparts, bioresorbable polymeric devices have a few advantages, including no requirement for a removal operation, no bone growth restriction related to gradual reduction in mechanical strength, lower risk of osteoporosis caused by stress-shielding, no tissue reaction due to metallic corrosion, and no generation of artifacts on computed tomography [1]. Many studies in the fields of orthopedics [2,3], craniofacial surgery [4,5], and oral/maxillofacial surgery [6,7] have been conducted to demonstrate the effectiveness of these materials. However, some problems remain concerning bioresorbable polymers, such as the need to achieve increased strength equal to that of natural cortical bone, the need to improve the degradation rates of polymers to reduce their persistence in the human body, and the need for bioactivity features (e.g., osteoconduction and bone-bonding ability). It is critical to develop a bioresorbable and bioactive material that can satisfy these requirements.

In 1971, the first bioresorbable material was reported for bone fixation in the surgical treatment of maxillofacial fracture [8]. A wide variety of biodegradable materials have since been explored to develop new resorbable systems for various biomedical applications. This has opened a new era of material research and supported a technological revolution in bioresorbable materials. Based on the structural characteristics of their components, existing bioresorbable materials can be divided into three principal “generations” with distinct physical, chemical, and biological properties.

The first generation bioresorbable materials include those composed of a single homopolymer, each of which has conspicuous limitations. Polyglycolic acid (PGA) was the first biodegradable polymer used in clinical practice. Despite its outstanding mechanical properties, there is little use of PGA in maxillofacial reconstruction due to its rapid degradation rate, formation of acidic degradation by-products, and limited solubility [9]. In the true first generation, poly-l-lactic acid (PLLA) is regarded as an established bioresorbable material because of its inherent biocompatibility, high mechanical properties, ease of processing, and availability from renewable natural sources (e.g., corn) [9]. However, PLLA is limited by its slow degradation rate, which leads to an inflammatory response in vivo [10,11]. Although the limitations of each polymer have made clinical application difficult, copolymerization in distinct proportions has yielded “second-generation” co-polymers, which have favorable features. Poly-lactide-co-glycolide (PLLA/PGA) is a notable representative of this generation. Its mechanical properties and degradation time can be modified based on the PLLA:PGA ratio [12]. For example, the resorption times of 50PLLA/50PGA, 75PLLA/25PGA, and 85PLLA/15PGA are 1–2 months, 4–5 months, and 5–6 months, respectively [10]. Although second-generation materials are superior to first-generation materials, neither possess bioactivity features (e.g., osteoconduction and bone-bonding ability). Hence, “third-generation” materials (composites of inorganic/bio-ceramic fibers or particles and organic polymers) were created to address the weaknesses of previous generations.

Uncalcined/unsintered hydroxyapatite and poly-l-lactide acid (u-HA/PLLA) is an epoch-marking third generation bioresorbable material that has been used to produce bioresorbable bone fixation devices since the early 1990s. Numerous in vitro [13,14] and in vivo [15,16,17] studies have demonstrated that u-HA/PLLA composite possesses many favorable features such as radiopacity, high mechanical strength, biocompatibility, bioresorbability, bone-bonding, and osteoconduction. First, osteoconductive ability and bone-bonding characteristics are unique features that make u-HA/PLLA superior to bioresorbable polymers. In an animal research study in 2019, Dong et al. [15] suggested that u-HA/PLLA material possessed osteoconductivity absent from PLLA material. Second, the physical properties of u-HA/PLLA are suitable for the fabrication of bone fixation devices. Several studies of the physical properties of u-HA/PLLA material have concluded that this composite satisfies the required initial physical strengths and maintains sufficient strength for bone healing [14]. However, Sukegawa et al. [16] reported that in two patients who received u-HA/PLLA devices, some material persisted at >5 years after surgery. In that study, no foreign-body reactions were observed, whereas another study described an inflammatory response from a u-HA/PLLA plate at approximately two years after surgery [17]. This implies that extended material persistence in the body is associated with a greater risk of complications. Thus, a more rapidly bioresorbable material is needed with similar bioactive/osteoconductive potential.

Recently, a new generative nanomaterial of uncalcined/unsintered hydroxyapatite and poly-l-lactide-co-glycolide (u-HA/PLLA/PGA) has been developed with the aims of preserving the advantages of existing material (in terms of bioactive/osteoconductive potential) and shortening the resorption time. Because the mechanical and degradation properties of co-polymer PLLA/PGA are presumed to be regulated based on the PLLA:PGA ratio [12], this new nanomaterial is expected to have properties superior to those of u-HA/PLLA. The manufacturer reports that the new nanomaterial has mechanical features similar to those of the previous material, while its decomposition and absorption period is approximately 2–3 years. However, the biocompatibility and bioactive osteoconductivity of this nanomaterial remain unclear. Therefore, studies of its bone regeneration ability are needed.

The bone reconstruction of the maxillofacial region is particularly complicated. Bone defects in this region may be caused by cancer resection, trauma, congenital malformations, progressive skeletal deformity, or orthognathic surgery. Because of the complex three-dimensional (3D) structural features, reconstruction of bone defects in the craniomaxillofacial skeleton can be difficult. Recently, u-HA/PLLA material has been confirmed as a feasible choice to reconstruct the maxillofacial region [15,16]. Accordingly, we expected that the new nanomaterial, which also contains u-HA, would possess similar favorable bone regeneration ability in the maxillofacial area.

In this study, we evaluated the bioactive/osteoconductive bone regeneration capacity and bioresorbability of u-HA/PLLA/PGA in the maxillofacial bone, compared with u-HA/PLLA materials, by implanting the materials to cover critical defects in rat mandibles. To our knowledge, this is the first animal study to assess the bone regeneration ability of this novel regenerative nanomaterial.

## 2. Materials and Methods

### 2.1. Materials

**Figure 1:**
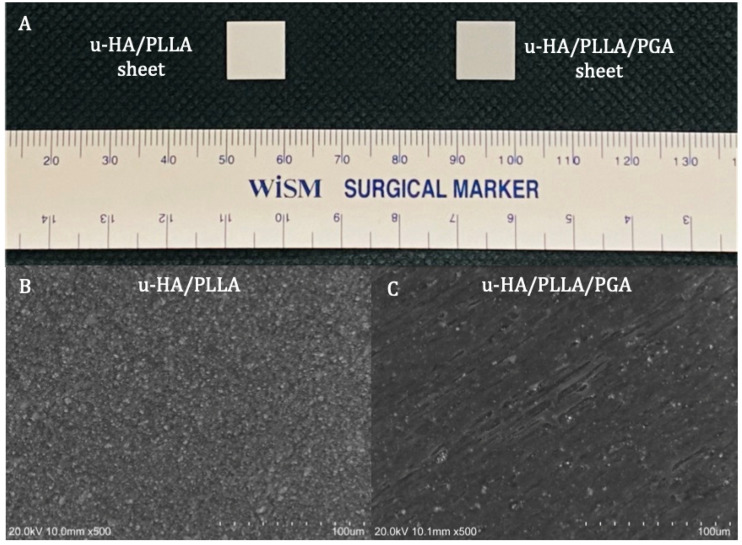
Materials. (**A**) Examples of a u-HA/PLLA (**left**) and u-HA/PLLA/PGA (**right**) sheets. (**B**) Scanning electron microscope image of u-HA/PLLA material. (**C**) Scanning electron microscope image of u-HA/PLLA/PGA material.

The two reconstructive materials used in this study were forged composite sheets of u-HA/PLLA/PGA (Teijin Medical Technologies Co., Ltd., Osaka, Japan) and u-HA/PLLA (Super Fixsorb-MX; Teijin Medical Technologies Co., Ltd., Osaka, Japan), both with dimensions of 10-mm length × 10-mm width × 0.3-mm thickness (Figure 1A). The u-HA/PLLA/PGA included 10% weight u-HA and 90% weight poly-L-lactide-co-glycolide, while the u-HA/PLLA consisted of 40% weight u-HA and 60% weight PLLA. In the co-polymer PLLA/PGA, the PLLA: PGA ratio was 88:12. The u-HA particle size ranged from 0.2 to 20 μm (mean size, 3–5 μm). The calcium:phosphorus molar ratio was 1.69 and the CO_3_^2−^ molar level was 3.8% (Figure 1B,C).

### 2.2. Surgical Procedure

**Figure 2:**
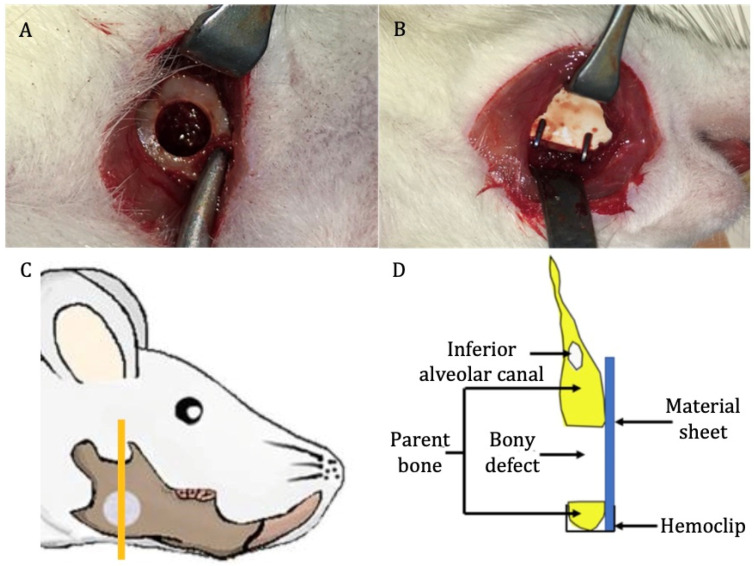
Surgical procedure. (**A**) Critical-size defect created at the mandibular angle on the right side. (**B**) Placement of reconstructive material. (**C**) Site of sample taken for analysis (the orange vertical line) (image modified from Sha et al. [18]). (**D**) Schematic coronal view of the specimen.

In total, 28 male Sprague Dawley rats (age = 10 weeks; weight = 250–270 g) were divided into three groups: u-HA/PLLA/PGA (*n* = 12), u-HA/PLLA (*n* = 12), and sham control (*n* = 4). Each group was divided into four subgroups of 1, 3, 8, and 16 weeks of follow-up time. At each time point, there were three rats in the u-HA/PLLA/PGA subgroup, three rats in the u-HA/PLLA subgroup, and one rat in the sham control group. All rats received general anesthesia via an intraperitoneal injection technique with medetomidine hydrochloride (0.15 mg/kg), midazolam (2 mg/kg), and butorphanol (2.5 mg/kg). All surgeries were performed under standard aseptic conditions. A 1-cm full-thickness longitudinal incision was created through the submandibular skin on the right side. The soft tissue was then dissected and retracted to expose the mandibular angle area. A 4-mm-diameter critical-size defect was created at the mandibular angle using a trephine bur from the buccal side to the lingual side (Figure 2A). Then, the defect was covered buccally with either u-HA/PLLA/PGA or u-HA/PLLA, as follows: each rat in the u-HA/PLLA/PGA group received one u-HA/PLLA/PGA sheet, whereas each rat in the u-HA/PLLA group received one u-HA/PLLA sheet. The sheets were fixed in place with hemoclips. In the sham control group, the defect was not covered (Figure 2B). The defect was then irrigated with normal saline and closed in layers. All rats awoke 1–2 h after surgery and showed normal behavior and appetite.

At 1, 3, 8, and 16 weeks after surgery, the rats were euthanized by anesthetic overdose. The right hemi-mandible was harvested and soaked in 10% neutral buffered formalin for further analysis (Figure 2C,D).

All animal experiments adhered to the Guidelines for Care and Use of Laboratory Animals of Shimane University Faculty of Medicine, Izumo, Japan. The animal protocol was approved by the Animal Ethics Committee of Shimane University (approval number: IZ 31-61).

### 2.3. Micro-Computed Tomography (CT) Evaluation of the New Bone Formation

High-resolution micro-CT was used to evaluate new bone formation in three dimensions. The rats’ right mandibles with the corresponding material sheets were scanned with a 3D Micro X-ray CT CosmoScan FX scanner (Rigaku Corporation, Tokyo, Japan) after animals had been sacrificed and before samples were sent for hematoxylin and eosin (HE) and immunohistochemical (IHC) staining.

**Figure 3:**
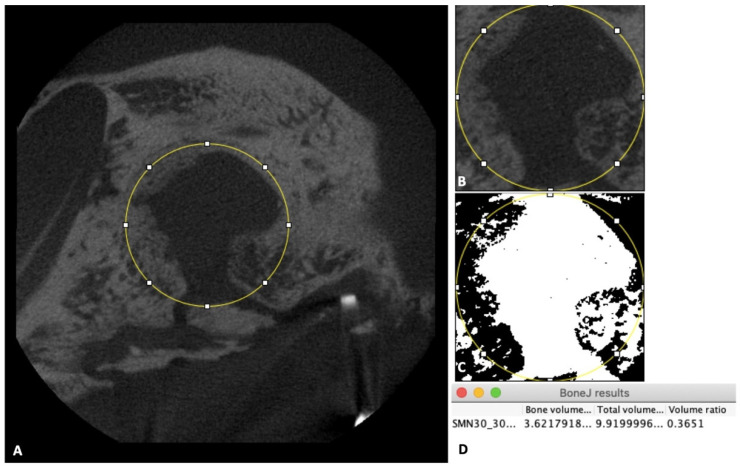
Micro-CT evaluation. (**A**) Image of the 4-mm-diameter circle including the initial defect and new bone inside the defect. (**B**) Illustration of the new 3D volume after using the “Duplicate” tool. (**C**) Illustration of the new 3D volume after using the “Make binary” tool. (**D**) Box shows final results.

Then, 3D volumes of the scanned samples were generated from acquired 2D lateral projections using Fiji software [19]. Before analysis, scanned bone volumes were digitally reoriented using the “TransformJ: Rotate” plugin in Fiji to achieve the correct axes of the defect. A 4-mm-diameter circle was drawn to represent the initial defect (Figure 3A). Next, a new 3D volume including only the initial defect and the new bone volume inside the defect was created using the “Duplicate” tool in Fiji software (Figure 3B). Finally, the results of bone volume, total volume, and volume ratio were calculated with the “Area/Volume fraction” plugin in Fiji software (Figure 3D) after using the “Make binary” function for the new 3D volume that had been created above (Figure 3C).

### 2.4. Tissue Preparation, HE Staining, and IHC Staining

#### 2.4.1. Tissue Preparation and HE Staining

The samples from each group at weeks 1, 3, 8, and 16 were decalcified, dehydrated, and paraffin-embedded. The specimens were sectioned along the coronal plane such that each final section included the defect area, the upper and lower parent bone, and the reconstructed sheet (Figure 2C,D). The sections were then stained with HE for histological evaluation and histomorphometry analysis.

#### 2.4.2. IHC Staining with Runx2, Osteocalcin (OCN), and Leptin Receptor (LepR)

The paraffin-embedded tissue specimens were cut into 4-μm sections. The sections were deparaffinized with xylene and rehydrated with ethanol. Enzymatic antigen retrieval was carried out using proteinase K (0.4 mg/mL). A 3% hydrogen peroxide solution was then used to block endogenous peroxidase activity. The sections were incubated with rabbit polyclonal anti-Runx2 or anti-LepR antibodies, or with mouse monoclonal anti-OCN antibody, for 50 min at room temperature. After sections had been washed three times with phosphate-buffered saline, they were incubated with Histofine Simple Stain MAX PO (MULTI) (#414191; Nichirei Biosciences Inc., Tokyo, Japan) for 30 min at room temperature. Finally, the sections were incubated with diaminobenzidine (DAB) for 10 min and counterstained with hematoxylin for 2 min. All IHC analysis procedures were performed by Sept. Sapie Co., Ltd. (Tokyo, Japan). The stained slides were observed using a BX43 light microscope (Olympus Corp., Tokyo, Japan).

### 2.5. Histomorphometric Evaluation

**Figure 4:**
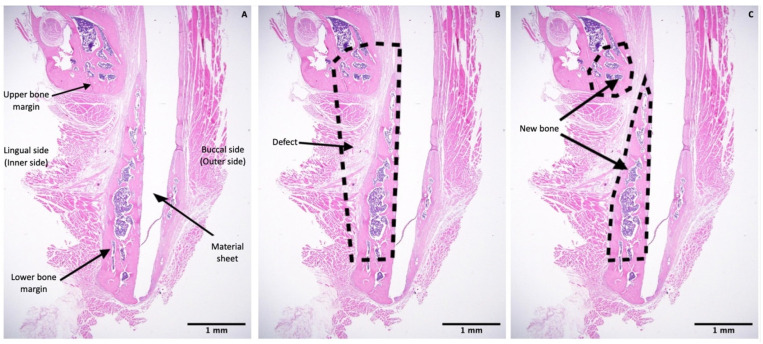
Histomorphometric evaluation. (**A**) Image at 1.25× magnification including the upper and lower bony margins of the defect region and the sheet covering the defect. (**B**) Illustration of the whole defect region. (**C**) Illustration of the new bone area in upper and lower bony margins. Scale bars: 1 mm (Black).

Histomorphometry was conducted to quantify the percentage of new bone formed within the defect region in a slide of each sample. One image at 1.25× magnification was taken for each slide using a high-resolution camera (Microscope Digital Camera DP21; Olympus Corp, Tokyo, Japan) mounted on the microscope. Each image included the whole defect region, the upper and lower bony margins of the defect region, the sheet covering the defect, and the lingual periosteum. Images were analyzed using a plugin in Fiji software [20]. The analysis method was similar to the approach used in a previous study [21]. Briefly, the total area as mentioned above (i.e., the whole defect) and the new bone area were selected using the selection tool in Fiji. These selections were saved using the region of interest (ROI) manager tool. Then, all areas were measured and the percentage of new bone was calculated using the formula: P = N/T × 100%, where P is the percentage of new bone area, N is the area of new bone area, and T is the area of the whole defect region (Figure 4).

### 2.6. IHC Assessment

**Figure 5:**
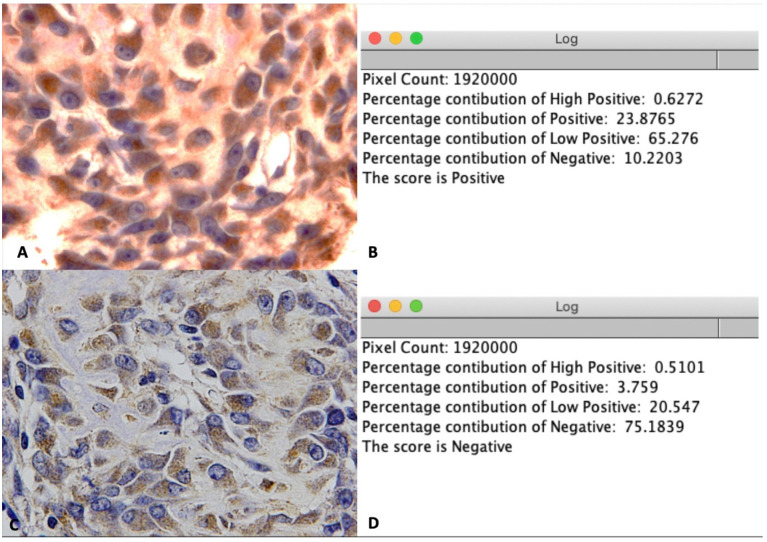
IHC OD score for LepR staining. (**A**) Illustration of a positive image at 100× magnification. (**B**) Positive score for image A after using the IHC profiler plugin in Fiji software. (**C**) Illustration of negative image at 100× magnification. (**D**) Negative score for image C after using the IHC profiler plugin in Fiji software.

Expression levels of Runx2 and LepR were quantified using IHC optical density (OD) score in accordance with the method of Jafari et al. [22] using the open-source plugin IHC profiler in Fiji software [19]. According to the Lambert–Beer law [23], the OD is relative to the staining concentration. The amount of stain present indicates the OD at a wavelength specific to the stain. On the basis of this theory, Ruifrok et al. [24] demonstrated that a color deconvolution algorithm could be employed on standard red, green, and blue (RGB) images to calculate the OD differences in Runx2 and LepR staining on each red, green, and blue channel in the RGB image. The IHC profiler plugin in Fiji software automatically calculated the OD of Runx2 and LepR via color deconvolution, in accordance with the algorithm provided by Ruifrok et al. [24]. The IHC profiler then automatically computed the semiquantitative OD score corresponding to the intensity of each pixel (Figure 5). Accordingly, three images at 100× magnification were taken at each of the following regions: the upper bone margin, lower bone margin, defect region adjacent to the reconstruction sheet, and center of the defect. Hence, 12 images were collected for each specimen. All images were then stored in TIFF format and analyzed using the IHC profiler plugin in Fiji software. Finally, for each image, an algebraic formulation was used to convert the semiquantitative outcome into a quantitative result to allow quantitative analysis and comparisons between groups, using the method of Bai et al. [25], as follows:

IHC OD score = 3 × H + 2 × P + 1 × L + 0 × N
H: percentage contribution of High positive
P: percentage contribution of Positive
L: percentage contribution of Low positive
N: percentage contribution of Negative

The mean IHC OD score of 12 pictures was calculated for analysis.

Because was deposited predominantly in the extracellular matrix of bony tissue [26], the expression of OCN in the new bone area was quantified using the digital H-score in accordance with the methods of Fuhrich [27], Nguyen [28], and Dong [15]. This method measures the intensity of the DAB chromogen stain using the intensity function in the Fiji software, where a higher intensity of DAB indicates a higher concentration of antigen. Numerically, a darker DAB signal will have a higher intensity and carry a lower value on a scale from 0 to 255. Hence, the three above studies suggest using the digital H-score (i.e., reciprocal intensity) to show the appearance of antigen.

**Figure 6:**
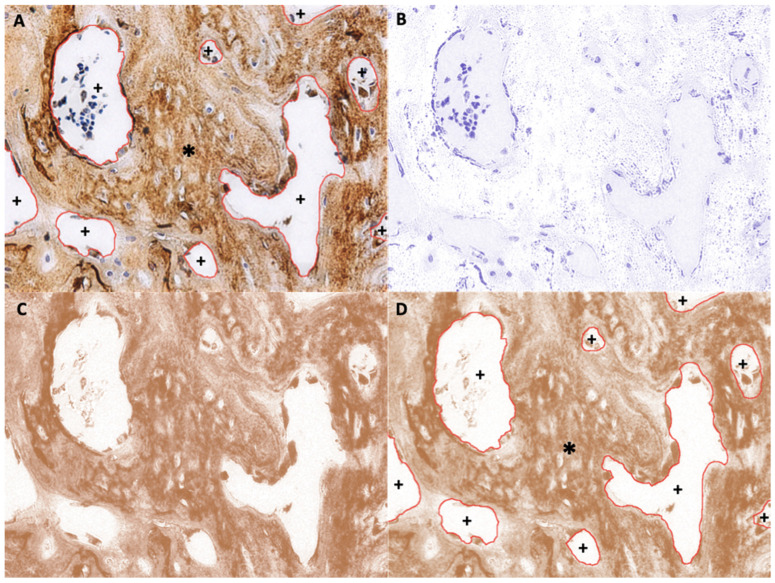
Example of new bone area ROI selection (anti-OCN IHC staining). (**A**) Original image with overlay of the selected ROI, which is the area of new bone. *, selected area; +, unselected area. (**B**) Hematoxylin-stained image separated from the original image. (**C**) DAB-stained image separated from the original image. (**D**) Superimposition of the saved ROI onto the DAB-stained image. *, selected area; +, unselected area.

To calculate the digital H-score, first, an empty area was chosen and its RGB values were examined. If the values were not near 255, the “Subtract Background” function was applied to repair the unsmooth background. Second, the ROI was selected to establish the area of new bone using various selection tools (Figure 6A). The selection was saved to the ROI manager. Third, the “Color Deconvolution” function with a vector of H DAB was used to separate the image into three panels representing hematoxylin staining (Figure 6B), DAB staining (Figure 6C), and background. Then, the previously selected ROI was superimposed onto the DAB image (Figure 6D). Finally, the “Measure” function was used to quantify DAB intensity (i), which ranged from 0 (black) to 255 (white) (Figure 6D). The digital H-score (i.e., reciprocal intensity) (f) of all new bone area in each specimen was then calculated using the formula f = 255 − i, as described by Nguyen et al. [28].

### 2.7. Statistical Analyses

Statistical analyses were performed using SPSS software for Mac OS (version 20.0; IBM Corporation, Armonk, NY, USA). The Mann–Whitney U test was conducted to compare the percentage of new bone (micro-CT and histomorphometry), IHC OD score (Runx2 and LepR), and digital H-score (OCN) between the u-HA/PLLA/PGA and the u-HA/PLLA groups at different time points. An intra-group comparison was also carried out. Values of *p* < 0.05 were considered to indicate statistical significance.

## 3. Results

### 3.1. Micro-CT Evaluation

**Figure 7:**
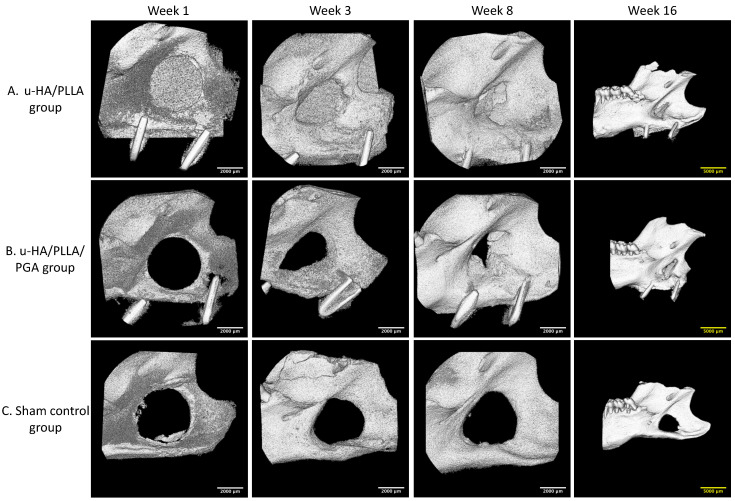
3D micro-CT images. (**A**) 3D views of the u-HA/PLLA group. (**B**) 3D views of the u-HA/PLLA/PGA group. Newly formed bone increased over time. (**C**) 3D views of the sham control group. New bone formation could not be identified. To present a broad view, images at week 16 are lower magnification than images at other weeks. Scale bars: 2000 μm (white), 5000 μm (yellow).

In 3D analysis, although the u-HA/PLLA sheets could be identified more easily than the u-HA/PLLA/PGA sheets because of the greater proportion of u-HA in the compound, the formation of new bone was comparable between groups. The newly formed bone inside the defect increased significantly from the beginning to the subsequent time points. The defect was filled largely at week 16 in both groups (Figure 7A,B). At each time point in the study, there were changes in the size of the 4-mm-diameter round-shape defects in the sham control group. This showed that the newly formed bone in this group was negligible (Figure 7C).

**Figure 8:**
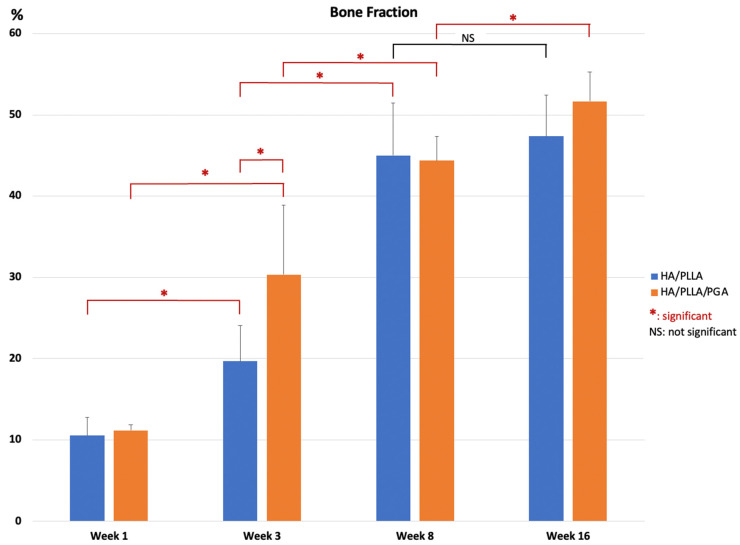
Percentage of new bone volume determined by micro-CT evaluation of u-HA/PLLA and u-HA/PLLA/PGA groups. * *p* < 0.05.

However, the amounts of new bone formation in the u-HA/PLLA and u-HA/PLLA/PGA groups at each time point were relatively homogeneous. The percentages of new bone volume in both groups were lowest at week 1 (10.52% and 11.16%, respectively), and then increased at weeks 3 (19.64% and 30.32%, respectively), 8 (45.00% and 44.38%, respectively), and 16 (47.48% and 51.66%, respectively). The tendency for increasing bone fraction was greater in the u-HA/PLLA/PGA group than in the u-HA/PLLA group from week 1 to week 16. At week 3, the percentage of newly formed bone in the defect was significantly greater in the u-HA/PLLA/PGA group than in the u-HA/PLLA group (*p* < 0.05). Nonetheless, there were no significant differences between the two groups at other time points. However, in each material group, the percentages of new bone formation differed significantly between weeks 1 and 3, and between weeks 3 and 8 (*p* < 0.05). Between weeks 8 and 16, there was only a significant difference in the u-HA/PLLA/PGA group (Figure 8).

### 3.2. Histological Evaluation

**Figure 9:**
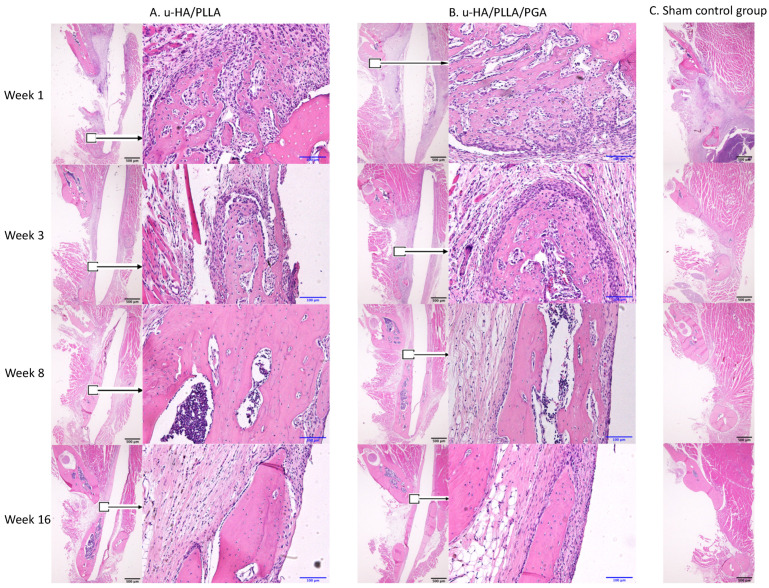
Hematoxylin and eosin-stained sections from the u-HA/PLLA, u-HA/PLLA/PGA, and sham control groups at weeks 1, 3, 8, and 16. Images in each subgroup were taken at 1.25× and 20× magnification (from left to right). Images of the sham control group were taken at 1.25× magnification. (**A**) u-HA/PLLA group. (**B**) u-HA/PLLA/PGA. (**C**) Sham control group. Scale bars: 100 μm (blue), 500 μm (black).

The u-HA/PLLA and u-HA/PLLA/PGA groups displayed similar histomorphometry characteristics at each time point. At week 1, bone spicules and newly formed blood vessels were observed at the defect–parent bone margins and the center of the defect. Numerous layers of osteoblastic cells were concentrated in the area around the bone spicules. Fibrous tissue appeared around the u-HA/PLLA and u-HA/PLLA/PGA sheets. At week 3, new bone formation from parent bone was evident in both groups. The new bone was mostly immature with a low level of mineralization and was surrounded by a large number of osteoblastic cells. The multilayered osteoblastic cells remained concentrated at the defect–bone margins. Fibrous tissue around the sheets was more easily identified, compared with analysis at week 1, and some new bone had grown from the parent bone near the sheets. At weeks 8 and 16, the presentations of bone formation in both groups were equivalent. A large amount of new bone with the lamellar bone pattern was observed at the center of the defect. The new bone showed features of maturity that were indistinguishable from the parent bone, including the presence of multiple osteocytes. Some new immature bone was formed in the tip of the mature new bone with several layers of osteoblastic cells. Fibrous tissue remained evident around the sheets, but steadily changed to bone tissue in the area near the defect–bone margin. It was clear that the direction of the new bone formation followed the axis of the material sheet and that the new bone always remained in contact with the surface of the sheet (Figure 9A,B).

In contrast, in the sham control group, no new bone was recognizable at the center of the defect. Migration of the masseter and the medial pterygoid muscle into the defect space from both sides was evident beginning in week 3 (Figure 9C).

### 3.3. Histomorphometry

**Figure 10:**
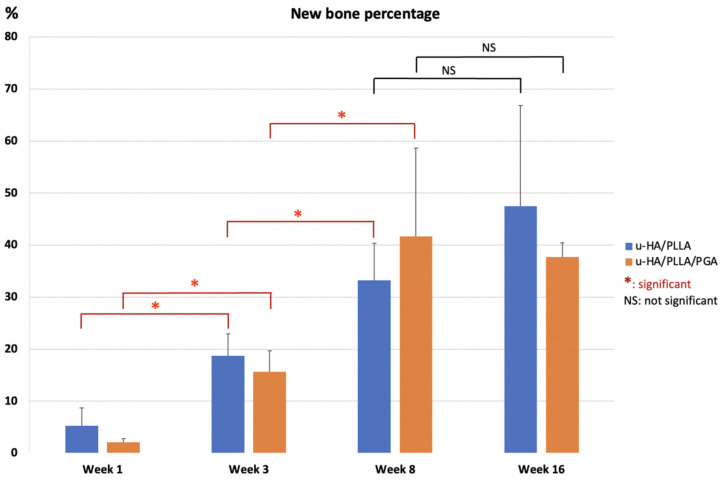
Percentages of new bone area in histomorphometric evaluation of u-HA/PLLA and u-HA/PLLA/PGA groups. * *p* < 0.05.

The mean percentages of the new bone area in the u-HA/PLLA and u-HA/PLLA/PGA groups at the center of the defect increased from week 1 to each of the subsequent time points. These results indicated no differences between the two groups among time points. However, in each group, the differences between weeks 3 and 1 and between weeks 8 and 3 were significant (*p* < 0.05). Although the mean percentages of subgroups at week 16 seemed to be higher than those at week 8, these differences were not significant (*p* > 0.05) (Figure 10).

### 3.4. IHC Analyses

#### 3.4.1. Runx2

**Figure 11:**
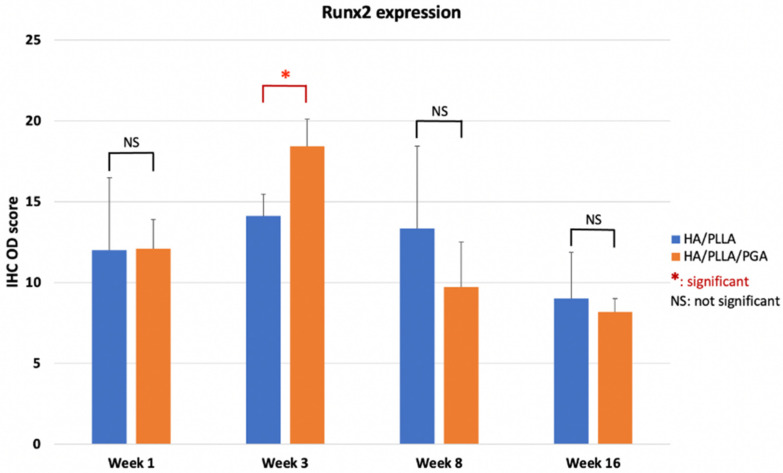
IHC OD score of Runx2 in the u-HA/PLLA and u-HA/PLLA/PGA groups. * *p* < 0.05.

**Figure 12:**
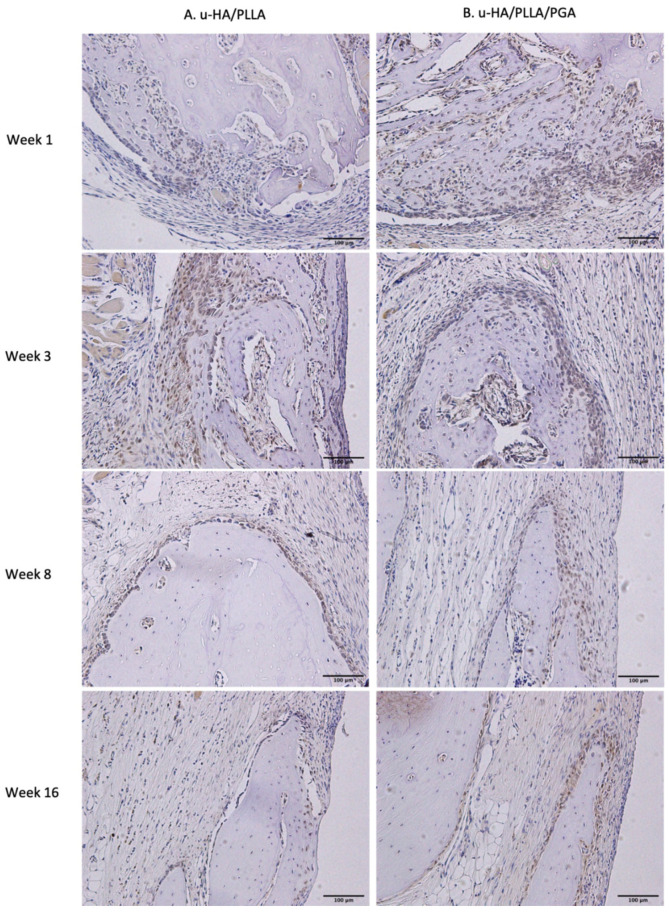
Runx2 expression in the u-HA/PLLA and u-HA/PLLA/PGA groups. All images were taken at 20× magnification. Runx2 expression was similar in the u-HA/PLLA and u-HA/PLLA/PGA groups. (**A**) u-HA/PLLA group. (**B**) u-HA/PLLA/PGA group. Scale bar: 100 μm (black).

The IHC OD score of Runx2 in the u-HA/PLLA and u-HA/PLLA/PGA groups increased from week 1 to week 3 in both groups (Figure 11). The Runx2-positive cells were congregated in the parent bone at the defect–bone margin and scattered in the center of the defect near the materials at weeks 1 and 3 (Figure 12A,B). Runx2 expression was highest at week 3 in both groups, although its expression was significantly higher in the new nanomaterial group than in the u-HA/PLLA group (*p* < 0.05). After week 3, the IHC OD score of Runx2 in both groups decreased gradually at weeks 8 and 16 (Figure 11). At these time points, Runx2 expression was mainly observed at the periphery of the new bone and the area near the materials (Figure 12A,B). Discrepancies between the two groups at weeks 1, 8, and 16 were not significant (*p* > 0.05).

#### 3.4.2. Leptin receptor

**Figure 13:**
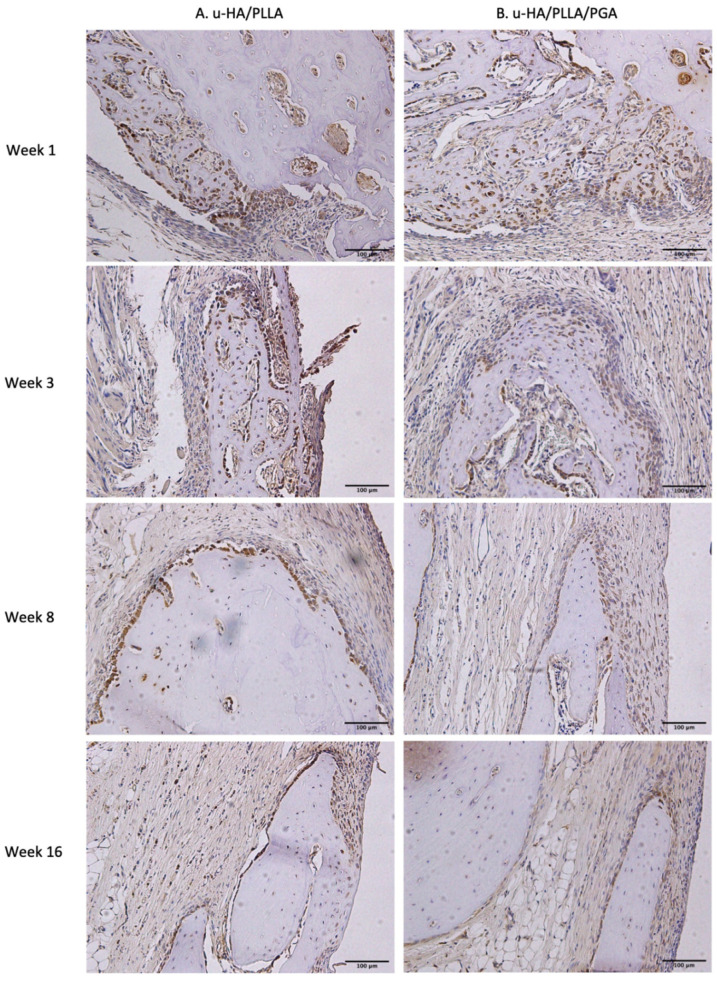
LepR expression in the u-HA/PLLA and u-HA/PLLA/PGA groups. All images were taken at 20× magnification. (**A**) u-HA/PLLA group. (**B**) u-HA/PLLA/PGA group. Scale bar: 100 μm (black).

Similar to the expression pattern of Runx2, LepR-positive cells were evident mainly in the parent bone at the defect–bone margin and scattered in the center of the defect near the materials at weeks 1 and 3, whereas they were near the periphery of the new bone and the area near the materials at weeks 8 and 16. Comparing images of LepR with images of Runx2, LepR-positive cells were observed in the same area and had an arrangement similar to that of Runx2-positive cells (Figure 13). Furthermore, a number of Runx2-positive cells also expressed LepR.

**Figure 14:**
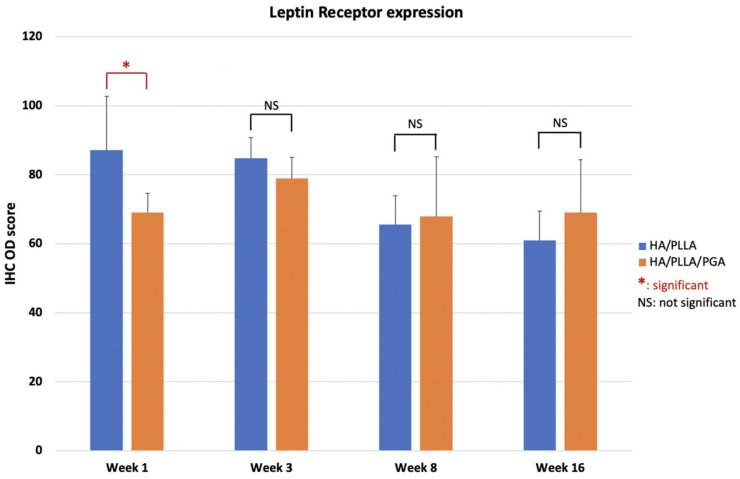
IHC OD score of LepR in the u-HA/PLLA and u-HA/PLLA/PGA groups. * *p* < 0.05.

LepR expression in the u-HA/PLLA group was highest at week 1, then decreased gradually at weeks 3, 8, and 16. In contrast, LepR expression in the u-HA/PLLA/PGA group was highest at week 3, while differences between week 3 and each other time point in this group were negligible (Figure 14). The difference between the two groups was significant only in week 1 (*p* < 0.05).

#### 3.4.3. OCN

**Figure 15:**
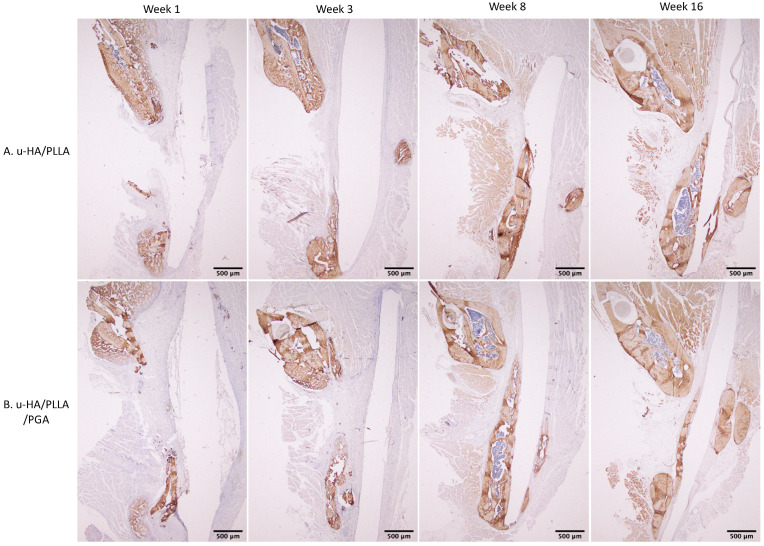
OCN expression in the u-HA/PLLA and u-HA/PLLA/PGA groups. All images were taken at 1.25× magnification. (**A**) u-HA/PLLA group. (**B**) u-HA/PLLA/PGA group. Scale bar: 500 μm (black).

The expression patterns of OCN were similar between groups. At weeks 1 and 3, low expression of OCN in the area near parent bone showed that the newly formed bone was immature. At weeks 8 and 16, the area of the defect in contact with parent bone had higher OCN expression. This indicated that the new bone had gradually matured (Figure 15).

**Figure 16:**
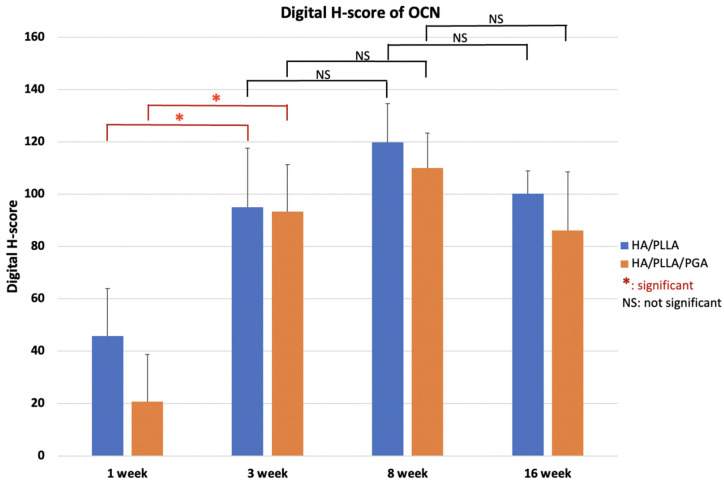
Digital H-scores based on IHC staining with anti-OCN antibody in the u-HA/PLLA and u-HA/PLLA/PGA groups. * *p* < 0.05.

The OCN expression levels in both groups were not significantly different between the two groups at various time points (*p* > 0.05). Expression was low at week 1, increased until week 8, then decreased slightly at week 16 (Figure 16). These results indicated that the differences between weeks 1 and 3 were significant in both groups (*p* < 0.05).

## 4. Discussion

### 4.1. Bone Regeneration Ability

To assess the bone regeneration capacity of u-HA/PLLA/PGA, histomorphometry and micro-CT methods were used in this study. In histomorphometry, the percentage of the new bone area was calculated in one cross-section of the defect (Figure 10), while in micro-CT, the results reflected the new bone volumes in three dimensions (Figure 8). Despite the use of two different methods for evaluation, the outcomes consistently indicated that the newly formed bone was similar in both u-HA/PLLA/PGA and u-HA/PLLA groups at all time points except week 3, when the percentage of bone fraction was higher in the u-HA/PLLA/PGA group than in the u-HA/PLLA group. These findings confirmed that our measurement results were unbiased.

Statistical analyses of the histomorphometry and micro-CT outcomes revealed that the bone regeneration abilities were comparable between materials and the amount of newly formed bone in the defect increased gradually at different time points. There were significant differences in the percentages of new bone between weeks 3 and 1 and between weeks 8 and 3 (*p* < 0.05). The mean amount of newly formed bone was greater at week 16 than at week 8, but this difference was not statistically significant. This implied that the important point for evaluation of new bone formation is around 8 weeks (Figure 8 and Figure 10).

Some studies have investigated the biocompatibility and osteoconductive properties of u-HA/PLLA. In an in vitro study in 1999, Shikinami et al. [14] analyzed the stoichiometric calcium phosphate molar ratio of deposited crystals using scanning electron microscopy and energy dispersive X-ray spectroscopy. They found that a layer of calcium phosphate crystals surrounding the material began to form on the composites after 3–6 days of immersion in simulated body fluid at 37 °C, then covered the whole surface with a fairly thick layer in 7 days. In an in vivo study in 2019, Sukegawa and colleagues [29] also investigated fibrous tissue surrounding u-HA/PLLA materials. Using histological assessment, they demonstrated that the uniform continuous fibrous tissue contained bone tissue and had no inflammation. They also suggested that because of this layer, u-HA/PLLA material has high biocompatibility and robust osteoconductive features. Additionally, according to Kokubo et al. [30], interactions between bioactive ceramics and the surrounding biological environment result in the formation of a calcium phosphate surface reaction layer and serum protein adsorption, in which this reaction layer adjusts the structure of the adsorbed serum protein (i.e., fibronectin). Specific bonding patterns within the molecules are then exposed to osteoblastic cells and their progenitors that are attached to the material surface. The cellular differentiation, bone matrix formation, and mineralization, which are regulated by the activation of intracellular signaling mechanisms, lead to bone bonding and a stimulatory effect on bone tissue formation. This clarifies the mechanisms of osteoconductive properties of both u-HA/PLLA and u-HA/PLLA/PGA materials. In 2019, Dong and colleagues [15] conducted an in vivo study on PLLA and u-HA/PLLA materials using a method similar to that of the present investigation. Their histomorphometry results showed a gradual increase in new bone formation within the defect. However, the mean new bone areas in the u-HA/PLLA group in their study were approximately 35% at week 4 and >60% at week 8, whereas this value reached only 33% at week 8 in our study. These dissimilarities were presumably because the material sheets in the study by Dong et al. [15] were attached closely to the buccal side of the mandible, while the material sheets in our study were unintentionally tilted laterally at the upper side. This tilt created an upward dihedral angle with two planes, which were the buccal side of the mandible and the lingual side of the material. Because of the reduced contact between the materials and mandible, the mean area of new bone was less in our study than in the study by Dong et al. [15]

**Figure 17:**
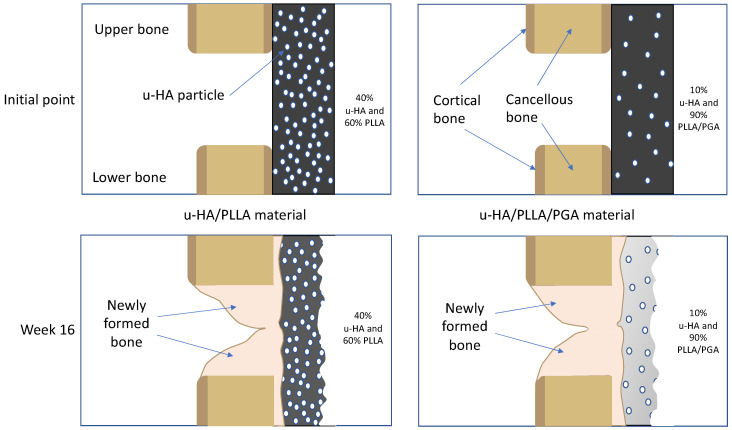
Theoretical explanation of the relationships between the bone remodeling capacity of u-HA/PLLA/PGA material and the u-HA particle proportion and degradation rate. Because of the more rapid degradation process, the u-HA particles in the new nanomaterial may be exposed to body fluids at an earlier stage.

As mentioned above, due to the presence of u-HA in the compound composition, u-HA/PLLA and u-HA/PLLA/PGA materials possess the capacity for bone regeneration. The proportion of u-HA in the new nanomaterial (10%) is considerably smaller than that in the previous material (40%). However, the results in the present study indicated that the bioactive/osteoconductive capacity of the new nanomaterial was comparable with that of the previous material. Notably, Runx2 expression in week 3 was significantly higher in the new nanomaterial group than in the previous material group (Figure 11). This implies that in the early stage, the bone remodeling capacity of u-HA/PLLA/PGA is higher than that of u-HA/PLLA material. This phenomenon might be caused by the more rapid degradation time of the new nanomaterial. As noted previously, the u-HA/PLLA material remains in the body at >5 years after surgery [16], while the manufacturer indicates that the duration of decomposition and absorption for the new nanomaterial is approximately 2–3 years. Because of the more rapid degradation process, the u-HA particles of the composites may be exposed to body fluids at an earlier stage. This promotes the differentiation of osteoblast cells and earlier bone regeneration in the defect (Figure 17). Therefore, despite its smaller proportion of u-HA particles, the new nanomaterial appears to stimulate bone formation more rapidly than the previous material in the early stage.

### 4.2. Bone Regeneration Pattern

In our study, HE staining revealed a fibrous tissue layer surrounding material sheets at week 1. This layer was evident at weeks 3, 8, and 16 (Figure 9). Sukegawa et al. [29] also reported that fibrous tissue was evident in three patients in their study. In both our study and the study by Sukegawa et al. [29], no inflammatory cells or giant cells against foreign bodies were apparent in the area of fibrous tissue. Thus, these materials exhibit biocompatibility. Furthermore, IHC analyses showed that Runx2 expression was highest at week 3, then decreased at weeks 8 and 16 (Figure 11). Some other studies reported similar changes in Runx2 expression based on IHC analyses [15]. Beginning in week 3, because most Runx2-positive cells were present at the tip of the parent bone near the fibrous tissue along with the material sheet, the newly formed bone covered the parent bone and remained in contact with the surface of the material. During new bone maturation, numerous Runx2-positive osteoblasts continued accumulating in the tip of the newly formed bone and the fibrous tissue. This process occurred repeatedly.

HE staining and micro-CT revealed that newly formed bone only grew from the parent bone and attached directly to the material at weeks 8 and 16. In a 2013 study, Moroi et al. [31] covered all aspects of the defect at the inferior border of the mandible by using a u-HA/PLLA membrane. After 1 week, the newly formed bone was directly concentrated on the u-HA/PLLA mesh surface without any parent bone contact. This phenomenon was contrary to our findings, presumably because only one side was covered in our study. Thus, all other sides of the defect were always in contact and filled with soft tissue, resulting in reduced bone regeneration. Therefore, at week 4, the new bone in the space between the parent bone and membrane occupied nearly 90% in the study by Moroi et al. [31], which was significantly higher than the results in the study by Dong et al. [15] and in our study. However, covering all aspects of the defect in the maxillofacial area is difficult in clinical practice because the bone structures in this area contain many thin walls and hollow structures, such as the paranasal sinuses or the orbits.

The distance from the material to the parent bone had a major influence on the amount and location of newly formed bone. In the histomorphometric evaluation, at week 8, the percentage of the new bone area of the u-HA/PLLA group was substantially higher in the study by Dong et al. [15] (61.77%) than in our study (33.24%). As mentioned above, in our study, the material sheets were unintentionally tilted toward the outside at the top of the defect, leading to an increased distance from the material to the parent bone on the top. Thus, more newly formed bone was observed on the bottom. In the non-ideal situation where only one aspect of the defect can be covered, closed contact between the bone and material is therefore essential.

The previous findings and our current results indicate that this fourth-generation new nanomaterial is suitable for reconstruction of the maxillofacial region, especially in the defects of the orbital wall and anterior wall of the sinuses (e.g., frontal and maxillary sinuses) due to trauma or elective tumor ablation. The main reason is presumably that the new nanomaterial possesses bioactive/osteoconductive features that may help to regenerate gaps between bone fragments at the fracture site where grafting with bone substitute is technically challenging. Second, the complete degradation process of u-HA/PLLA/PGA is approximately 2–3 years, which is adequate for full recovery of the bony defect and eliminates the need for material removal. The total resorption time is still relatively long but is shorter compared to the resorption time of 5.5 years for the previous third-generation bioresorbable bioactive/osteoconductive material (u-HA/PLLA) as the short duration of bioresorbability helps to avoid the complication of an inflammatory response in the body. Finally, to regenerate the bone in the defect area, the bone regeneration pattern in this study indicates that the material only must closely contact bone near the defect on one side without requiring complete coverage. Thus, u-HA/PLLA/PGA has the potential to become a “fourth-generation” bioresorbable material,

### 4.3. Immunohistochemical Representation of Runx2 and LepR Expression

Runx2 is the main regulator of osteoblast differentiation [32,33,34] and serves as a determinant of bone remodeling and skeletal integrity functions. Additionally, Zhou et al. [35] showed that LepR, a receptor for a fat-cell-specific hormone expressed in approximately 0.3% of bone marrow cells, is an excellent marker for the prospective identification of mouse mesenchymal stem cells. LepR-positive cells are normally quiescent, but proliferate after an injury to regenerate bone [35]. Yang et al. [36] found that LepR-positive cells include two populations: LepR^+^Runx2-GFP^low^ (64.6% ± 2%) and Lep+Runx2-GFP^−^ sub-populations. Importantly, LepR^+^Runx2-GFP^low^ cells differentiated into mature osteoblasts through multilayered cell formation with increasing levels of Runx2 expression. In our study, the presence of Runx2-positive cells and LepR-positive cells in IHC analyses revealed that multilayered cells were identical in the edge of the parent bone (Figure 12 and Figure 13). This observation implied that bone marrow could be a main source of osteoblastic cells. Furthermore, Runx2- or LepR-positive cells were observed to concentrate on fibrous tissue in contact with parent bone. The fibrous tissue around these composites was considered a favorable environment for bone regeneration, consistent with the findings in some previous studies [14,15,29]. Finally, the observations in IHC analyses and the results of Runx2 and LepR expression confirmed that the osteoconductivity of u-HA/PLLA/PGA was similar to that of u-HA/PLLA.

### 4.4. Potential Weaknesses of New Material

During micro-CT assessment, we noticed that the u-HA/PLLA material was radiopaque and could be easily detected by the naked eye, whereas the u-HA/PLLA/PGA material was radiotransparent (Figure 7). Because the weight proportion of u-HA particles in u-HA/PLLA/PGA materials is only 10%, it is difficult to observe these sheets in micro-CT or conventional X-ray images. This indicated that the radiologic evaluation of this new nanomaterial after surgery could be difficult.

### 4.5. Limitations of This Study and Future Prospects

First, because our study was conducted in a rat model, our results are not representative of human patients. Second, because of ethical concerns, the number of animals in our study was low, which may have led to biased results. Therefore, to determine whether this novel nanomaterial is feasible for surgical applications, additional validation studies are needed.

Further studies are required to examine neoangiogenesis and vascularization in the bony defect region, as well as the infiltration of new blood vessels into the regenerated bone and the body of the material itself. The mechanical properties of the newly regenerated bone should also be assessed.

## 5. Conclusions

The results obtained in this study imply that the newly developed u-HA/PLLA/PGA bioresorbable nanomaterial may accelerate bone regeneration more rapidly than the third-generation u-HA/PLLA bioresorbable material during the early bone healing stage. The bone regeneration ability of u-HA/PLLA/PGA material was comparable or better than that of u-HA/PLLA material in the rat mandible defect model. Moreover, contact between the parent bone and the bone regenerating material could be critical for clinical success. These findings show that this new nanomaterial, u-HA/PLLA/PGA, has great potential for clinical application as a fourth-generation bioresorbable material.

## Data Availability

All data have been illustrated in the manuscript.
